# Scarlet Fever

**Published:** 1900-12-29

**Authors:** Percival Mackie

**Affiliations:** Resident Medical Officer, Bristol City Isolation Hospitals, Ham Green


### Hospital Clinics and Medical Progress.

### SCARLET FEVER.

By Percival Mackie, M.R.C.S.Eng., L.R.C.P.Lond.; Resident Medical Officer, Bristol City Isolation Hospitals, Ham Green.

(*Continued from page* 206.)

The rash is liable to many variations according to the colour of the skin and length of time it has been "out." The general blush appears first and goes first; the skin may be dotted with injected papillæ for 24 hours after the erythematous element has disappeared. In brunettes the rash is apt to be dark and indefinite and the red points less marked. In severe cases the rash is often very dark, deeply set and patchy, and sometimes associated with minute hæmorrhages. In well-marked cases of moderate severity, especially in blondes, the subcuticular blush may be so brilliant that the whole skin is in a vivid scarlet flush without separate elements. In these cases there is often a very fine powdery desquamation present, giving an appearance aptly described as being like "powder and red rouge." On the legs, and often to a less degree on the arms, the elements are quite discrete, and the rash is in papules, macules, or spots, and often in vesicles. There is a special variety in which the whole body is covered with small vesicles, each overlying an injected papilla: this is called the "miliary" rash, and the individual vesicles are almost exactly like sudamina. The rash may last only a few hours, but more generally for two or three days; and in some severe cases we have seen a vivid rash persist for over a fortnight.

*Desquamation.*—Desquamation is the physical result of the rash, and depends upon it for its intensity and duration. It generally commences a day or two after the rash has subsided, but sometimes it is delayed for three weeks. It is most slight and soonest over in infants and small children, and remains longest where the skin is thickest and driest. One kind of desquamation has already been referred to as occurring concurrently with the rash where the latter is very intense. The true desquamation of scarlet fever is only seen in other conditions associated with a *punctate* rash; a simple erythema is never followed by the kind of desquamation to be described. It commences over the site of each papilla of the skin as a minute shining branny scale which gradually separates and drops off leaving a small circle: these circles extend into the surrounding neighbourhood, and beginning by being pin-head in size become ultimately the size of a threepenny or sixpenny piece, thus meeting other circles. In between these circles so formed there remain irregular islets of desquamation which separate in flakes on the hands and feet. The skin is too hard for these processes to occur, and here when the skin is horny (especially therefore in adults) the skin come off in flakes and even in glove-like casts sometimes of the whole hand at once.

*Sore Throat.*—This symptom is almost constant, but the degree is very variable. It is convenient for purposes of description to make five separate types, according to the intensity and extent of the morbid processes, just as in surgery one speaks of five degrees of burns. This is not a theoretical division only, but one which is borne out by actual practice.

First Degree.—In this, the mildest variety, there is injection of the whole pharyngeal structures without swelling, exudation, or ulceration, and it is unaccompanied by pain or glandular enlargement.

Second Degree.—Here the tonsils, soft palate, &c., are deeply injected, swollen, and velvety, the tonsils approximate to one another, there is some discomfort or actual pain on swallowing, and the cervical glands are often slightly enlarged and tender.

Third Degree.—This is a more severe type than the last, and besides the changes described in the second degree there is considerable exudation met with, overlying the inflamed surfaces, but still without breach of surface. The exudation is in the form of lymph and muco-pus lying over the parts in strands and hanging in festoons from side to side. It is associated with a sticky, dirty tongue, and the cervical glands are enlarged, often to the eye, and tender; suppuration rarely follows. Nourishment is taken with pain.

Fourth Degree.—In this variety there is ulceration of the tonsils, almost invariably on their opposing surfaces, and pus is formed and collects with thick mucus in the throat. There is generally associated post-nasal inflammation, with consequent mouth breathing, and therefore generally the tongue is dry and caked, and the lips and teeth are sometimes covered with sordes. The cervical glands are often much enlarged, and suppuration sometimes follows. Otorrhœa is a common complication. Sometimes it is only with difficulty that the child can be induced to take nourishment.

Fifth Degree.—This type of sore is so severe that it gives rise to the name scarlatina anginosa. It is a very severe type, and a large proportion of the fatal cases die from this cause. It is usually associated with severe rhinitis and otorrhœa, and extensive ulceration and sloughing in the pharynx. In several cases we have found, after death, ulceration extending from the posterior nares down to the base of the epiglottis, perforation of the soft palate, complete sloughing away of a tonsil, suppuration around the laryngeal cartilages, and in some cases extensive ulcerative stomatatis. These are the worst cases one sees in fever hospitals: the child passes into an asthenic condition and dies, generally within a week, of toxæmia, œdema of larynx, or, if he lives long enough, of septicæmia. Now and again we see throats which can scarcely be placed under any of these headings: one of such is like a follicular tonsillitis with discrete plugs of secretion in the tonsillar crypts. In another class there is a thin veil overlying one or both tonsils, not opaque like a true membrane, but semi-transparent. We have never found Klebs Loëffler bacilli in this throat, nor do the clinical symptoms suggest true diphtheria.

*Circulatory System.*—The poison of scarlet fever has a marked effect upon the heart, firstly by causing disturbance of the pulse-temperature ratio; and, secondly, by its tendency to produce peri-, myo-, or endo-carditis. It is quite common to find a pulse rate of 120 associated with a temperature under 100° F., and in young children particularly a pulse rate of 160, 180, or even 200 is not necessarily a serious symptom taken by itself.

Again, endocarditis is in our experience not very uncommon if the presence of a whiffing mitral systolic murmur audible in the axilla and an enlarged left ventricle be regarded as evidence of such. These signs usually disappear in a fortnight or three weeks; but it is easy to understand that chronic valvular disease may in some cases result. In the present series of cases no instance of pericarditis has occurred, but some maintain that it is more frequent than endocarditis. In a few cases the heart becomes rapid and slightly dilated without any sign either of peri- or endo-cardial changes: these cases are very obstinate, and may remain quite unaffected by digitalis, strychnine, and other drugs. The pulse rate remains between 100–130, and is apt to undergo great variation in a short time from nervous influences; in fact, the heart is irritable. As an instance, the case of an adult man whose pulse rate taken by reliable nurses was always about 96 but taken by myself was generally 120–130, varying during successive minutes between 100–130 simply from "nervousness." These signs point to the presence of myocardial changes, though we have never had the opportunity of verifying the statement by microscopic examination. In one or two cases these signs having persisted for three or four months, and in spite of rest in bed and medicinal treatment, the patient has been discharged uncured.

*(To be continued.)*

